# Lessons learned from health system rehabilitation preparedness and response for disasters in LMICs: a scoping review

**DOI:** 10.1186/s12889-024-17992-2

**Published:** 2024-03-14

**Authors:** Justine Gosling, Roxanne Maritz, Ariane Laplante-Lévesque, Carla Sabariego

**Affiliations:** 1 Center for Rehabilitation in Gobal Health Systems, Faculty of Health Sciences and Medicine, University of Lucerne, Lucerne, Switzerland; 2 Department of Behavioural Sciences and Learning, Linköping University, Linköping, Sweden; 3 Swiss Paraplegic Research, Nottwil, Switzerland; 4 Faculty of Health Sciences and Medicine, University of Lucerne, Lucerne, Switzerland

**Keywords:** Disasters, Health emergencies, Health systems, Preparedness, Rehabilitation

## Abstract

**Introduction:**

Disasters such as earthquakes, conflict, or landslides result in traumatic injuries creating surges in rehabilitation and assistive technology needs, exacerbating pre-existing unmet needs. Disasters frequently occur in countries where existing rehabilitation services are underdeveloped, hindering response to rehabilitation demand surge events.

**Aims:**

The primary aim of this scoping review is therefore to synthesize the evidence on rehabilitation and assistive technology preparedness and response of health systems in LMICs to the demand associated with disasters and conflict situations. A secondary aim was to summarize related recommendations identified in the gathered literature.

**Methodology:**

A scoping review was conducted using the Arksey and O’Malley framework to guide the methodological development. The results are reported in accordance with PRISMA-ScR. Four bibliographic databases were used: CINHAL, Cochrane, Pubmed, Scopus and. Key international organisations were also contacted. The search period was from 2010–2022. Eligible publications were categorized for analysis under the six World Health Organization health systems buildings blocks.

**Results:**

The findings of this scoping review suggest that rehabilitation is poorly integrated into health systems disaster preparedness and response in LMICs. Of the 27 studies included in the scoping review, 14 focused on service delivery, 6 on health workforce, 4 on health information systems and 3 on the leadership and governance building block. No study focused on financing nor assistive technology. This review found the most frequently referenced recommendations for actions that should be taken to develop rehabilitation services in disasters to be: the provision early and multi-professional rehabilitation, including the provision of assistive technology and psychological support, integrated community services; disaster response specific training for rehabilitation professionals; advocacy efforts to create awareness of the importance of rehabilitation in disasters; and the integration of rehabilitation into disaster preparedness and response plans.

**Conclusion:**

Findings of this scoping review suggest that rehabilitation is poorly integrated into health systems disaster preparedness and response in LMIC's, largely due to low awareness of rehabilitation, undeveloped rehabilitation health systems and a lack of rehabilitation professionals, and disaster specific training for them. The paucity of available evidence hinders advocacy efforts for rehabilitation in disaster settings and limits the sharing of experiences and lessons learnt to improve rehabilitation preparedness and response. Advocacy efforts need to be expanded.

**Supplementary Information:**

The online version contains supplementary material available at 10.1186/s12889-024-17992-2.

## Background

The outcome of conflict and other disasters such as earthquakes or landslides frequently result in traumatic injuries such as fractures, burns, amputations, spinal cord and crush injuries, among others, creating surges in rehabilitation and assistive technology needs [1]. This surge need can quickly overwhelm the health system’s ability to provide rehabilitation, delaying urgent interventions or making accessing services difficult or impossible. [2–4]. There can be significant and life changing consequences for those whose rehabilitation needs are unmet or delayed, leaving a legacy of disability for years to come [5–8].

The World Health Organization (WHO) defines rehabilitation as “*A set of interventions designed to optimise functioning and reduce disability in individuals with health conditions in interaction with their environment*" [9] and considers rehabilitation as a key element of universal health coverage [10] as well as an essential component of emergency response [1, 6]. Further cementing its status as an essential health service, the 2023 landmark World Health Assembly rehabilitation resolution EB152/10 calls for the timely integration of rehabilitation into emergency preparedness and response plans.

The vital role and benefits of rehabilitation are well recognized in global rehabilitation disaster and emergency response guidelines from the highest authorities in health [11–14]. Early rehabilitation in disaster contexts has been shown to reduce disability and improve quality of life [7, 15, 16]. Longer term, rehabilitation is a wise societal investment, supporting individuals to participate in family life, education, and employment [9].

Conflict and other disasters frequently occur in low- and middle-income countries (LMICs) where existing rehabilitation services may be underdeveloped [3, 17], due to a lack of resources and understanding of rehabilitation and its benefits. Without existing infrastructure and integrated services, these health systems are unlikely to have rehabilitation disaster preparedness frameworks, and therefore are not well positioned to respond to increased rehabilitation demand caused by surge events [18]. In such circumstance, those in need of rehabilitation services often do not receive them, or accessing services is delayed and only available at a time and place that is inadequate to meet the need [19].

The timeliness of delivery, quality and effectiveness of rehabilitation interventions are greatly enhanced when preparedness plans are in place prior to any disaster, to provide a framework for initiating necessary considerations and actions [20, 21]. The United Nations state that “*preparedness refers to the knowledge and capacities developed by governments, professional response and recovery organisations, communities and individuals to effectively anticipate, respond to, and recover from, the impacts of likely, imminent or current hazard events or conditions*” [22].

Countries such as Iran and Nepal have had disaster rehabilitation frameworks in place before a disaster occurred [8, 23]. However, evidence to evaluate LMICs health system rehabilitation disaster preparedness plans and frameworks to effectively respond to the impact of disasters with rehabilitation is scattered. An overview of the evidence is therefore essential to understand the current situation, learn from experiences, and develop consolidated recommendations that can inform the development of national disaster rehabilitation frameworks. This scoping review attempts to answer the research question of “What can we learn on rehabilitation preparedness and response in health systems in low- and middle-income countries in the context of conflict and disasters?” The primary aim of this scoping review is therefore to synthesize the evidence on rehabilitation and assistive technology preparedness and response of health systems in LMICs to the demand associated with disasters and conflict situations. A secondary aim is to summarize recommendations about rehabilitation and assistive technology service provision in disasters and conflict identified in the gathered literature.

For the purpose of this study, only disasters that may result in trauma were included. Such disasters include earthquakes, tropical storms, tsunamis, volcanic eruptions, fires, explosions, building collapse, floods, conflict, terrorism, and mass casualty incidents. In this study, LMICs are defined according to the World Bank 2022 classification [24].

### Methods

A protocol was developed by the research team guiding the literature search, publication selection, information extraction and descriptive synthesis of results. The Arksey and O’Malley framework [25] guided the methodological development. To ensure methodological quality and transparency, the results are reported according to the Preferred Reporting Items for Systematic Reviews and Meta-Analyses extension for Scoping Reviews (PRISMA-ScR) checklist [26]. See Additional file 1: Appendix 1 for details.

### Search strategy

For each concept relevant to the research question, a list of search terms was developed (Table 1). The Medical Subject Headings (MeSH) database was utilized to ensure all appropriate search terms were included and, to narrow the search and to improve specificity. Field terms were used when key terms were not included under the MeSH headings. Four bibliographic databases were used as they are among the largest and most authoritative medical journals relevant to rehabilitation: CINHAL, Cochrane, Pubmed and Scopus. See Additional file 2: Appendix 2 for details. A hand search of the reference lists of all eligible studies was completed to identify relevant publications not identified by the database searches.

**Table 1:** Key concepts and their search terms

Key concepts	Final search terms
Rehabilitation	Rehabilitation, rehabilitation and burn, wounds and injuries
Assistive products/ technology	Assistive product, Assistive technology, assistive product, assistive technology devices, self-help devices
Disaster	Disaster, humanitarian, humanitarian response, humanitarian crisis, humanitarian intervention, humanitarian action, armed conflict, natural disaster, disaster and rehabilitation, relief work

To consider grey literature such as reports or guidance documents from key stakeholders that have not been published in scientific journals or indeed their websites, 18 rehabilitation lead or focal persons working at key international organisations were contacted by email requesting relevant publications. This was considered an important step by the authors as these organizations are working in the field on the research topic and may provide eligible documents not publicly available. The organisations were: the Christian blind mission, European Physiotherapy Association, Humanity & Inclusion, the International Committee of the Red Cross, Interburns, International Society for Prosthetists and Orthotists, the International Society of Physical and Rehabilitation Medicine, Médecins Sans Frontières, World Physiotherapy, World Federation of Occupational Therapy, and WHO. Rehabilitation lead or focal persons were asked to respond within two weeks, and one reminder email was sent. Additionally, their organisations’ websites were searched for relevant publications.

### Eligibility criteria

Eligible articles should fulfil the following inclusion criteria:

- mention to propose, implement or evaluate the delivery of rehabilitation and/or assistive technology services in disaster or conflict situations.
- have a health systems perspective.
- focus on LMICs, as defined by the World Bank [24].
- be a primary research study, government or organisation policy paper or report.
- have been published between 2010 and 2022 (2010 was chosen as first year for the eligible period this is when the Haiti earthquake occurred).
- have been published in English and the full text was available.

Articles were excluded if they:

- were publications on pandemics, such as COVID-19 and Ebola, as these result in different short and long term rehabilitation needs on a much greater scale and are outside the scope of this review.
- were publications focusing on international emergency medical teams, military or short-term non-governmental organisations projects as these are not a part of a country’s government financed health system.
- reported refugee interventions in high-income countries.
- were publications in non-disaster or non-conflict situations.
- were a scoping or systematic review, commentary or opinion articles from foreign teams or book chapters and conference presentations.
- reported only about psychological rehabilitation, as this is specializing on a single in-depth topic beyond our searches scope.
- emergency preparedness for persons with disabilities, such as preparing for evacuations and medications.

Additionally, methodologically poor studies describing conclusions that cannot be considered reliable, or only mentioning rehabilitation but not giving details, were excluded. Studies reporting prevalence information for health systems, such as prevalence of people requiring rehabilitation following a disaster, were only included if information was collected through a health system relevant survey, such as those conducted by an official body of the health system such as the ministry of health.

### Eligibility assessment

The four authors working in the field of policy and research on topic of rehabilitation in health systems were involved in the screening process (JG, RM, ALL, CS). Studies obtained from the database searches were uploaded to EndNote, a reference manager tool, and duplicates were removed. The remaining studies were then uploaded to Rayyan, a literature review platform, [27], for eligibility assessment. JG reviewed the titles and abstracts of all articles against the inclusion criteria twice. A fifth reviewer, blinded to the first author’s decisions, reviewed 20% of the publications which were randomly selected from each year within the search period. A reconciliation meeting was held to discuss disagreements and agree upon the status of those studies with RM as moderator. The full text of the resultant 217 studies was read and assessed for eligibility by JG, and the final 27 included studies agreed upon by all authors.

### Data extraction and synthesis of results

The extracted data included study characteristics such as year of publication, disaster event, country, aims, type of study, participants, findings regarding their intervention, activities or observations on rehabilitation and/or assistive technology in disaster preparedness or response, as well as any recommendations the authors made. JG categorized included articles into one of the six WHO rehabilitation health system building blocks [28] based on the studies’ main focus to clearly present the evidence to answer the research question. The six building blocks are: service delivery, health workforce, health information systems, medical products such as assistive products, financing and leadership and governance. ALL reviewed the final categorization decisions, and disagreements were resolved in a reconciliation meeting. It is recognized that the building blocks are interlinked into health systems, and that eligible studies may touch on more than one block [29].

## Results

**Fig. 1:**
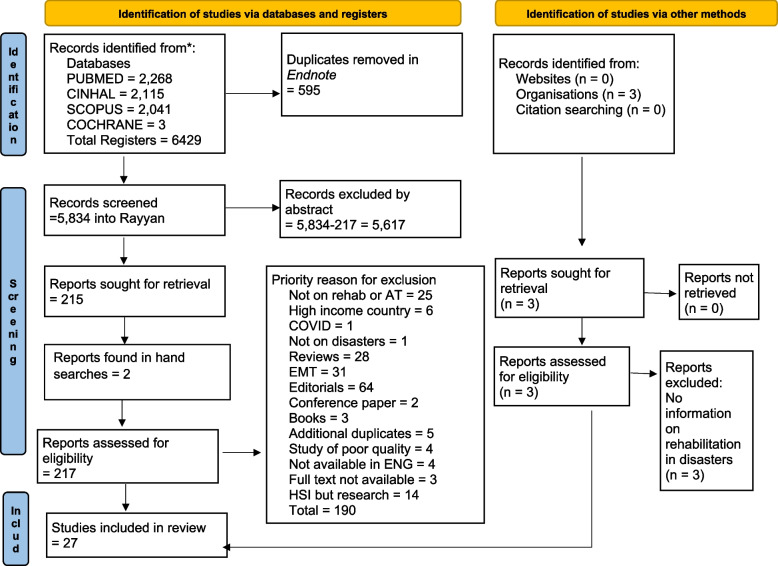
PRISMA 2018 flow diagram [26]

The database searches resulted in 27 studies for anlaysis (Fig. 1). The grey literature search and the contacting of key organisations focal persons yielded zero additional publications. Of the 18 individuals contacted, 7 replied by either sending their organisation’s global guidelines, or by apologizing that their reports were unpublished and for internal use only. Three national Systematic Assessment of Rehabilitation Situation (STARS) reports were identified from the website searches, but these were excluded as they did not provide information on rehabilitation services in conflict or disaster situations.

### Overview of included studies

The included studies were based on events in 11 countries: 9 from China, 4 from the Philippines, 2 each from Bangladesh, Haiti, India, Iran, and Nepal, and 1 each from Brazil, Nigeria, Pakistan and Turkey. Seventeen of the 27 studies described activities related to earthquakes, 6 were not focused on a specific disaster event, 2 were each related to landslides and typhoons. The majority of the 27 studies were observational studies or case reports, and many collected data years after the event.

When classified according to the six building blocks of health systems, 14 studies focused on service delivery, followed by 6 on health workforce, 4 on health information systems and 3 on the leadership and governance building block (Table 2, 3, 4 and 5). No study primary focused on the financing and assistive technology building blocks, however, these topics were mentioned in some of the studies that were categorized in other building blocks.

**Table 2:** Summary of the service delivery results

Title	Aims	Country	Disaster	Type of study	Participant group	Findings
Philippine academy of rehabilitation medicine emergency basic relief and medical aid mission project (November 2013-february 2014): The role of physiatrists in super typhoon Haiyan [3]	To describe the emergency basic relief and medical aid missions performed by physiatrists in response to Super Typhoon Haiyan	Philippines	2013 typhoon Haiyan	Case report	Unknown number of rehabilitation doctors	Besides providing medical care, physiatrists functioned as mission team leaders, as community advocates, and other roles. Services included free consultation and treatment; medicines, and wound care supplies to 7255 patients, which included non-disaster related care
Physical rehabilitation in the context of a landslide that occurred in Brazil [30]	To investigate the challenges in delivering rehabilitation to those injures in the 2011 landslide disaster	Brazil	2011 landslide	Cross-sectional mixed method study	2326 hospital records and 27 interviews with 11 victims and 16 health professionals	Most rehabilitation services didn’t identify a surge in demand post disaster, despite knowing demand existed. This was thought to be because the need was repressed by competing personal needs, financial constraints to pay for rehabilitation and transport, and lack of access and awareness of rehabilitation services, meaning referrals weren’t made
The outcomes and impact of a Post-Earthquake Rehabilitation Program in China: A Qualitative Study [31]	To analyze the outcomes and implications for a large-scale, community based, post-earthquake rehabilitation program in Sichuan, China after the program had been operational for 5 years	China	2008 Sichuan earthquake	Embedded qualitative case study	1,471 people who received rehabilitation services between July 2008 and June 2013	75.4% patients sustained injuries related in the earthquake, and the remaining 24.54% were non-earth-quake victims. 88.06% of service users felt the programed helped them achieve their goals. The program achieved favorable results in enhancing functional independence in activities of daily living, physical status and psychosocial well-being of the service users. The program has been transferred to the local partner, with some changes
Mobility, prosthesis use and health related quality of life of bilateral lower limb amputees from the 2008 Sichuan earthquake [32]	To report the rehabilitation outcomes (mobility, prosthesis use and quality of life) of bilateral lower limb amputees from the Sichuan Earthquake	China	2008 Sichuan earthquake	Observational cross sectional	17 bilateral lower limb amputees sustained in the earthquake	Results suggested that amputation level and knee joint salvage, prosthesis use and exercise were associated with better rehabilitation outcomes including mobility, adjustment and quality of life 9 amputees (52.9%) used wheel chairs, 2 (11.8%) used crutches,2 (11.8%) used walking sticks and 4 (23.5%) were unaided walkers
Morbidity pattern and impact of rehabilitative services in earthquake victims of Kashmir, India [33]	To know the nature of the injuries, magnitude of disability, rehabilitative services provided, and service satisfaction	India	2005 Kashmir earthquake	Retrospective observational study	266 earthquake injured patients needed surgical intervention	12.33% of patients had spinal injuries and 32% of all those injured received assistive products, with 90% still in use at 1 year follow up. 97% of patients felt rehabilitation services were beneficial
Epidemiology and the impact of early rehabilitation of spinal trauma after the 2005 earthquake in Kashmir, India [34]	To describe the epidemiology of spinal injury in the Kashmir Earthquake and to analyse the impact of 15 days of rehabilitation	India	2005 Kashmir earthquake	Descriptive observational case report	38 spinal cord injury patients	Provision of free assistive devices was the main rehabilitation intervention to prevent further spinal injuries. Psychotherapy and physiotherapy is important to maintain joint range and prevent contracture, and was deemed beneficial in the sample. 15 days of rehabilitation is not enough
Developing a trauma critical care and rehabilitation hospital in Haiti: A year after the earthquake [35]	To describe project Medishare which provides rehabilitation services and training to Haitian healthcare professionals	Haiti	2010 earthquake	Descriptive observational case report	NA	Daily patient care has been managed by Haitian medical staff as well as more than 2,400 international volunteers Efforts continue for building workforce capacity, and the priority is training and education of the workforce
Amputations of limbs during the 2005 earthquake in Pakistan: A firsthand experience of the author [36]	To audit the incidence of amputations of limbs in the 2005 Pakistan earthquake and their rehabilitation management	Pakistan	2005 earthquake	Retrospective descriptive study	112 patients with amputations of upper and lower limbs	Amputations accounted for 0.9% of total injuries and needed immediate rehabilitation for physical, psychological and occupational challenges which included prosthetic limb fittings
2017 Bangladesh landslides: physical rehabilitation perspective [37]	To describe the impact on the population affected by the 2017 landslides in south-eastern Bangladesh	Bangladesh	2017 landslide	Observational	Landslide survivors	Rehabilitation for traumatic injuries was limited due to a lack of staff trained in rehabilitation. Rehabilitation capacity building requires significant cooperation between academic medical institutions and other emergency response stakeholders
The NHV rehabilitation services program improves long-term physical functioning in survivors of the 2008 Sichuan earthquake: A longitudinal quasi experiment [6]	To quantify the effectiveness of the NHV rehabilitation program as measured by the function of earthquake survivors	China	2008 Sichuan earthquake	A longitudinal quasi–experimental design with an intervention group and control group	510 earthquake survivors	The NHV rehabilitation services significantly improved estimated Barthel index in both the early and late intervention groups compared to controls, demonstrating benefit from rehabilitation delivered nearly 1.5 years after injury
Functional outcomes and health-related quality of life in fracture victims 27 months after the Sichuan earthquake [38]	To evaluate functional outcomes, health-related quality of life and life satisfaction in fracture victims 27 months after the 2008 Sichuan earthquake	China	2008 Sichuan earthquake	A cross-sectional quasi-experimental study with 2 intervention groups and a control group	390 fracture victims divided into early or late intervention groups, or a routine care control group	Activities of daily living and life satisfaction in the intervention groups were significantly improved compared to the control group
Continuous post-disaster physical rehabilitation: a qualitative study on barriers and opportunities in Iran [39]	To outline the barriers and opportunities of disaster rehabilitation services in Iran	Iran	2003 Bam and 2012 Varzaghan earthquake	Observational	16 rehabilitation service providers, users and administration in the affected area of the two earthquakes	The main barriers to delivering disaster rehabilitation were found to be: decision makers low knowledge of rehabilitation, a lack of an effective responsible body; weak disaster-related competencies; and under-prioritization by government. Distribution of assistive devices was critical in affected areas Rehabilitation specialists could play a role in triage and proper immobilization of injured limbs
Evaluation of functional outcomes of physical rehabilitation and medical complications in spinal cord injury victims of the Sichuan earthquake [40]	To characterize spinal cord injuries from the 2008 Sichuan earthquake and evaluate their functional outcomes following rehabilitation	China	2008 Sichuan earthquake	A prospective observational cohort study	51 spinal cord injured earthquake victims from 3 hospitals	Ambulation, wheelchair mobility and ADL were significantly improved with rehabilitation. 35.3% of patients achieved at least moderate activity of daily living independence and 90.2% regained some self-care ability prior to discharge. The group that began rehabilitation more than 3 months after the earthquake did not show significant functional improvement
Responding to the health and rehabilitation needs of people with disabilities post-Haiyan [41]	To describe the activities to increase access to rehabilitation for people with disabilities and with injuries post-Haiyan	Philippines	2014 typhoon Haiyan	Descriptive field investigation report	2998 individuals needing rehabilitation and assistive devices	50 prostheses and 320 mobility aids were provided to people with new injuries or pre-existing disabilities. Having detailed pre-disaster data of estimations and profiles of people with disabilities would have improved the response
Factors affecting functional outcome of Sichuan earthquake survivors with tibial shaft fractures: a follow-up study [42]	To analyse the functional recovery of earthquake survivors with tibial shaft fractures in Sichuan, China	China	2008 Sichuan earthquake	Observational	174 ambulatory earthquake survivors with tibia shaft fractures	Functional recovery was positively associated with rehabilitation intervention (odds ratio 5.3 (95% confidence interval 2.38–11.67)

**Table 3:** Summary of the workforce results

Title	Aims	Country	Disaster	Type of study	Target group	Findings
Preparation, roles, and responsibilities of Filipino occupational therapists in disaster preparedness, response, and recovery [43]	To describe the roles, responsibilities and work of Filipino occupational therapists in disaster management	Philippines	Non-specific	Descriptive cross-sectional	24 occupational therapists with experience working in disasters	The roles most frequently performed were encouraging social interactions among survivors, providing mental health services to survivors and attending trainings in disaster response
Knowledge, practices and perceived barriers of physiotherapists involved in disaster management: a cross-sectional survey of Nigeria-based and trained physiotherapists [7]	To investigate the knowledge, practices and perceived barriers regarding the role of physiotherapists in disaster management among Nigeria-based and trained physiotherapists	Nigeria	Non-specific	Descriptive cross-sectional	50 registered physiotherapists with at least 1 year of work experience	68.7% of physiotherapists acknowledged their potential role in disaster management, but only 6.7% had experience, with 90% citing a lack of established government policies on the integration of physiotherapists into disaster management as a barrier. Involvement of physiotherapy during disasters is limited by financial, workforce, equipment, training, awareness and resource constraints
Rehabilitation nurses’ opinions on disaster rehabilitation services, their training needs and perceptions of preparedness for disasters [44]	To assess rehabilitation nurses’ perceptions of disaster preparedness and response, and to identify rehabilitation nurses’ training needs	Turkey	Non-Specific	Descriptive cross-sectional	50 female rehabilitation nurses	Participants mostly agreed that rehabilitation nurses have a role in disaster response, but 72% of them had no experience of disaster work and 94% felt that they need training in disaster rehabilitation. 90% were eager to receive training on this topic
Occupational therapy role in disaster management in Bangladesh [45]	To investigate the numbers and role of occupational therapists who have worked in disaster management in Bangladesh	Bangladesh	Non-specific	Observational	3 occupational therapists who had disaster response experience	There are very few OTs working in disaster management in Bangladesh. The 3 participants who respond reported undertaking preparedness activities such as community risk mapping, advocacy and providing assistive technology
The Urgent Rehabilitation Technique Education Program for Wenchuan earthquake [46]	To describe the activities of the specialist forum for the patients injured in the earthquake	China	Wenchuan 2008 earthquake	Case report	1500 hospital workers in the most earthquake prone areas	The short-term program developed covered the basic clinical technical trainings in physiotherapy, occupational therapy, prosthetics and orthotics to rapidly upscale rehabilitation service capacity for earthquake victims
Physiotherapy in Haiti: A qualitative study exploring local clinicians' perspectives [47]	To describe the strengths, weaknesses, opportunities and threats to the development of physiotherapy Haiti after the 2010 earthquake	Haiti	2010 earthquake	Descriptive study	4 physiotherapists and 1 rehabilitation technician	Respondents identified the lack of funding to be the main the profession's main barrier: funding to create employment opportunities for rehabilitation professionals, and for the population to be able to access affordable physiotherapy services

**Table 4:** Summary of the health information systems results

Title	Aims	Country	Disaster	Type of study	Target group	Findings	Function
Impairment and functional status of people with disabilities following Nepal earthquake 2015 [48]	To investigate the disability status of earthquake survivors a year after the earthquake	Nepal	2015 earthquake	Observational cross-sectional survey	29 persons with disability in the Bahunepati area	The average percentage score of disability, calculated by the WHODAS 2.O scoring guidelines was an average of 56%. One year after the earthquake, the number of people with disabilities was few, but the level of disability among them was high	Evaluation
Epidemiological analysis of trauma patients following the Lushan earthquake [49]	To analyze the earthquake injury characteristics and treatments	China	2013 Lushan earthquake	Observational study	2010 patients admitted to hospitals with earthquake related injuries	70.5% patients had limb dysfunction. For 60% of these, rehabilitation records could be found and the median time to start rehabilitation was 1 week and the median duration was 3 weeks. 508 patients required assistive technology devices	Implementation
Rehabilitation needs of the survivors of the 2013 Ya'an earthquake in China [50]	To determine the physical, functional and psychosocial rehabilitation needs of those injured	China	2013 Ya’an earthquake	Observational survey	143 survivors with lower limb and spinal fractures	74.8% required rehabilitation, 44.8% needed splints and 45.5% needed home modifications. There was a high need for assistive devices and home and community modifications due to environmental barriers or earthquake damage	Implementation

**Table 5:** Summary of the leadership and governance results

Title	Aims	Country	Event	Type of study	Participants	Findings	Function
Responding to physical and psychological health impacts of disasters: case study of the Iranian disaster rehabilitation plan [51]	To report the process of developing a comprehensive pre-disaster plan for physical and psychological rehabilitation Iran	Iran	Non-specific	Case report	80 health disaster experts working in 34 governmental and nongovernmental organizations	Sharing information, education, workforce training and funding were identified as the best methods of improving stakeholders’ participation and collaboration in formulating a disaster plan Inadequate basic services with unqualified staff had the greatest negative impact	Policy
The role of physical therapists in the medical response team following a natural disaster: Our experience in Nepal [8]	To describe the PT role in the response, and recommendations for future planning	Nepal	2015 earthquake	Case report	Nepal Physiotherapy Association	The immediate activation of the pre-established rehabilitation subcluster played a key role in coordinating the earthquake response and implementing a long-term rehabilitation strategy that included community services for those in remote locations, or who had lost their homes. Coordination and strong leadership are essential at all levels. National associations are well placed to support both national and local planning	Policy
Development of a national occupational therapy disaster preparedness and response plan: the Philippine experience [52]	To describe the process to produce the national occupational therapy Philippine disaster preparedness and response plan and to document the challenges of the task	Philippines	Non-specific	Descriptive report	Occupational therapists, community development workers and organizations of persons with disabilities	A national workshop took place and a disaster response plan and framework were produced to highlight the role occupational therapists should play in disaster response, and plans made to incorporate them into disaster response and to build up workforce capacity	Policy

### Service delivery

Most studies (*n* = 15) focused on rehabilitation service delivery (Table 2). This is an expected finding, considering that an individual’s rehabilitation need can only be met with direct service provision, and disaster and conflict situations create demand. Of these, 11 studies were based on earthquakes, with 6 of these from earthquake prone China. Nine studies evaluated service delivery and 6 reported the implementation of services. Most studies made recommendations to develop and integrate rehabilitation into emergency preparedness and response.

Mousavi et al. (2019) [53] iterated what other studies included in this scoping review also emphasized, that the delivery of rehabilitation services during disasters is highly dependent on the existing system, and that, in the absence of strong and integrated rehabilitation services within the existing health system, service delivery during disasters will be inadequate. Disasters also add to the existing unmet need for rehabilitation, with studies in the review reporting a large proportion of individuals with pre-disaster need accessing rehabilitation services set up for disaster victims [3, 31].

Taking Iran as an example, Mousavi et al. (2019) [53] reported that a lack of policy guidance, limited decision makers’ limited knowledge of rehabilitation, a lack of an effective responsible rehabilitation body; weak disaster-related competencies; and under-prioritization of rehabilitation by government were the greatest barriers towards developing rehabilitation services.

Describing reasons for a lack of rehabilitation services in disasters, Carvalho et al. (2019) [30] posed that low demand for services can occur, not because the need does not exist, but because they are not accessible and because rehabilitation need can be repressed by competing personal needs, financial constraints to pay for services and transport, and lack of access and knowledge of the rehabilitation services available.

Uddin et al. (2021) [37] suggested that a lack of rehabilitation professionals and disaster specific training were the main barriers to providing rehabilitation services for those with injuries sustained in the 2017 Bangladesh landslide. The authors recommended increasing rehabilitation capacity through task sharing and integrating rehabilitation technical training into the emergency response structure. Supporting this suggestion, Hotz et al. (2012) [35] recommended a train-the-trainer model to expand workforce capacity and capabilities, based on experiences in Haiti after the 2010 earthquake.

Ali et al. (2010) [33], Keshkar et al. (2014) [34], Li et al. (2019) [32], and Mousavi et al. (2019) [39] evidenced the long-term need for assistive technology provision for those injured in earthquakes, and emphasized that AT provision, as part of rehabilitation interventions, are associated with outcomes such as better functioning and greater quality of life. The case report from the Philippines by Ganchoon et al. (2018) [3] demonstrated that rehabilitation services can be effectively delivered within other relief and medical aid missions. Table 6 summarizes the recommendations from studies focusing on service delivery, with the top four most referenced recommendations being: 1) Early multi-professional rehabilitation [3, 32–34, 38, 40, 42]; 2) Assistive technology provision [3, 32–34, 39]; 3) Community-based rehabilitation provision [3, 38, 40], and; 4) Psychological support [33, 34, 42].

**Table 6:** Summary of recommendations from the service delivery studies

• **Early multi-professional rehabilitation ** [3, 32–34, 38, 40, 42]
**• Assistive technology provision** [3, 32–34, 39]
**• Community based rehabilitation provision ** [3, 38, 40]
**• Psychological support ** [33, 34, 42, 52]
• Effective pain relief [7, 38]
• Use of social media and patient education sheets to raise awareness of rehabilitation services available [3]
• Undertaking of an active search for people in need of rehabilitation and actions to ensure services are accessible [7, 30]
• Close collaboration between trauma surgical services and rehabilitation services [33, 44]
• Rehabilitation should be available with victim triage, assessment, at the scene, in district facilities, in mobile units and in hospitals [37]
• Home adaptations and other environmental barrier modifications, if needed [7, 40]
• Close relationships with local and international stakeholders to integrate rehabilitation response and improve future disaster responses and the allocation of resources [3]
• Expansion of workforce capacity and capabilities is essential [39], a train the trainer [35] model and task shifting [37] should be considered
• Special consideration and provision should be made for vulnerable populations or underserved rural areas to enable a rapid response [39–41]
• Empower and improve the rehabilitation capacity of the local community when designing a disaster response rehabilitation program [31]
• A professional volunteer recruitment database can hasten response [6]
• Organization needs to come from authority at the national level and advocacy work is needed to realize this [39, 47]
• Pre-disaster mapping of those who will need specific disability and rehabilitation services [41]

### Workforce

Six studies focused on rehabilitation workforce (Table 3) from 6 countries, 1 responding to an earthquake and the others not based on a specific disaster. The included studies demonstrate that the rehabilitation workforce has little experience in disaster preparedness and response. This was reported to be due to a lack of training and awareness of professionals in their role in disaster responders.

Habib et al. (2014) [45] found only three occupational therapists in Bangladesh who had disaster response experience, and these were from national or international non-governmental organizations. From the national occupational therapy professional organizations register, Ching et al. (2019) [43] found only 24 occupational therapists with disaster response experience in the Philippines, who mostly only had experience in providing mental health support to the pediatric population in disasters. These findings by Ching et al. (2019) [43] contradict an earlier policy study by Duque et al. (2013) [52], classified in this review under the leadership and governance category, describing the process and challenges faced to produce the national Philippines disaster preparedness and response plan for occupational therapists. In the aforementioned plan, key recommendations to support workforce development in disaster preparedness and response were made, which, 5 years on, appeared to have been minimally implemented.

The included articles link the lack of rehabilitation workers’ experience in disasters to them having little understanding of the role they can play. In a Nigerian survey, Ojukwu et al. (2019) [7] found that only 68.7% of physiotherapists acknowledged their potential role in disaster management. Conversely, in the only eligible study involving rehabilitation nurses, Kalanlar et al. (2021) [44] surveyed nurses in a single hospital in Turkey and found that most felt they had a role to play in disaster response. However, 72% of the nurses had no experience of disaster work, and 94% felt that they need training in disaster rehabilitation.

He et al. (2011) [46] described the implementation of a program to teach basic rehabilitation skills to rapidly increase rehabilitation capacity for earthquake victims, and advocated for its use in future emergencies. However, the programs outcomes, costs, implementation challenges, and sustainability were not described. Table 7 summarizes recommendations of studies focusing on workforce with the top 3 most referenced recommendations listed foremost: 1) entry level and post graduate practical training to develop disaster management knowledge and skills [7, 43, 44, 46]; 2) professionals and their organisations should be involved in shaping disaster policy and advocacy [7, 45, 47], and 3) creating an awareness of the role of rehabilitation disaster among the public and other healthcare professionals [7, 43, 47].

**Table 7:** Summary of recommendations from the workforce studies

**• Entry level and post graduate practical training to develop disaster management knowledge and skills** [7, 43, 44, 46]
**• Professionals and their organizations should be involved in shaping disaster policy and advocacy ** [7, 45, 47]
**• Create an awareness of the role of rehabilitation disaster among the public and other healthcare professionals** [7, 43, 47]
• Focus on rural areas and services in primary health and community care [7, 47]
• Establishing of government policies on the integration of rehabilitation in disaster management [7]
• Funding to employ rehabilitation workers in government facilities [47]
• Mental health support training [43]

### Health information systems

Three studies from 2 countries fulfilled the criteria for the health information systems category (Table 4), all of them relating to earthquakes. The studies provided data demonstrating that disasters can result in significant disability [48], long term need for rehabilitation and assistive technology, and community modifications due to environmental barriers or earthquake damage [49, 50].

However, the quality of the eligable studies design and sampling was poor and as a result, likely underrepresents true prevalence. For example, a poorly described and sampled study by Bimali et al. (2018) [48] excluded people who have difficulties with understanding and communicating, co-morbidities, or preexisting disability. The study by Zhang et al. (2014) [49] collecting data following an earthquake acknowledges that many patients received treatment outside the province, and therefore their data could not be included. Table 8 summarizes the recommendations of studies focusing on health information systems, with the most referenced recommendation being that provision of early and appropriate rehabilitation, which includes psychological support and assistive technology, is essential.

**Table 8:** Summary of recommendations from the included health information systems studies

• **Provision of early and appropriate rehabilitation with psychological support ** [48–50]
**• Assistive technology and splints must be provided for ** [49, 50]
• Focus on vulnerable populations needing specialist care, such as the elderly and children [49]
• Community functional rehabilitation services [50]
• Education and advocacy on the role of rehabilitation [50]
• Capacity for patient home visits, environmental modifications and equipment [50]

### Leadership and governance

Three policy studies from Iran, Nepal and the Philippines were categorized under leadership and governance (Table 5), all documenting professional organization’s attempts to develop national rehabilitation disaster preparedness plans. These studies suggest that disaster preparedness and response activities are driven from the ‘bottom up’ of a health system in the absence of policy, and indicate a possible lack of awareness of rehabilitation in disasters at national coordination levels.

Ardalan et al. (2016) [51] described the process of developing a comprehensive disaster preparedness plan for rehabilitation in Iran. The authors identified that information sharing, advocacy in the media, workforce education, and availability of funding were the best methods for improving stakeholders’ participation and collaboration in formulating a rehabilitation disaster plan. Inadequate basic services provided by unqualified staff had the greatest negative impact on formulating a rehabilitation disaster response plan. Reflecting on the 2015 earthquake, the Nepal physiotherapy association [8] made recommendations for future disaster response planning. They reflected that the immediate activation of the pre-established rehabilitation subcluster played a key role in coordinating the earthquake response, and also in implementing a long-term rehabilitation strategy that included community services for those in remote locations or who had lost their homes. They emphasized that coordination and strong leadership are essential for effective response, and that professional associations should be consulted. Table 9 summarizes recommendations of studies focusing on leadership and governance with the most referenced recommendations listed being: 1) Advocacy on the role of rehabilitation professionals [8, 51, 52] and 2) rehabilitation professionals and community health workers should be involved in drafting of disaster response plans [8, 51, 52].

**Table 9:** Summary of recommendations from the leadership and governance results

• **Advocacy on the role of rehabilitation professionals** [8, 51, 52]
**• Rehabilitation professionals and community health workers should be involved in drafting of disaster response plans ** [8, 51, 52]
• Government policy makers should be involved in formulating rehabilitation disaster plans to ensure integration of rehabilitation into overall disaster response [51, 52]
• Acute trauma rehabilitation should be incorporated into undergraduate rehabilitation courses, with a particular focus on spinal injuries and amputees, triaging of patients, first aid, and; application of plaster casts [8, 52]
• Each organisation should be given extensive pre and post disaster tasks to avoiding misunderstandings and improve coordination [51, 52]
• A system should be developed to evaluate the implementation any rehabilitation plan, using valid and reliable indicators [51]
• Early establishment of a rehabilitation subcluster to support disaster response [8]
• General systems strengthening is imperative [8]
• Allocation of funds for disaster training [52]

## Discussion

This scoping review synthesised the evidence on the preparedness of health systems in LMICs to respond with rehabilitation services and assistive technology to the demand associated with conflict and disasters situations. Additionally, we collated the recommendations identified in the gathered literature. The body of published literature was found to be small with just 27 eligible studies, and their findings demonstrate that integration of rehabilitation in disaster response and preparedness in LMICs is limited across all health systems building blocks. Studies focusing on service delivery were the most cited for all the building blocks, perhaps a result of requests for evaluation and achievement reporting.

Importantly, the results are not indicative of a lack of the need for rehabilitation and assistive technology in disasters, but rather the lack of published literature in English on the topic, or a lack of access to it. It has been suggested that the scarcity of literature highlights the gap between service delivery and evaluation of interventions, and that further research is needed on measurement strategies, accountability mechanisms, and patient-centered approaches in humanitarian settings [54]. The paucity of evidence hinders advocacy efforts for rehabilitation in disaster settings and limits the sharing of experiences and lessons learnt to improve rehabilitation preparedness and response.

The substantial number of studies (*n* = 17) published on earthquakes could be due to the large media and political attention earthquakes receive, possibly making them more attractive for researchers and easier to gain funding and support for. Most studies addressed service delivery, what is expected considering that rehabilitation need requests service provision, and disaster and conflict situations create demand. Additionally, our searches indicate that there is a lack of published data collected on the need for rehabilitation in disasters, and consequently, there is a lack of evidence to support investing resources and initiating actions to develop rehabilitation response plans. The four most prominent recommendations were: the provision of early and multi-professional rehabilitation, including assistive technology, psychological support, and integrated community services; disaster response specific training for rehabilitation professionals; advocacy efforts to create awareness of the importance of rehabilitation in disasters, and; the integration of rehabilitation into disaster preparedness and response.

### Service delivery

The literature categorised under service delivery stressed the vital role of early rehabilitation and long-term rehabilitation and assistive technology needs for victims, while underlining that emergency provision can only be successfully provided if developed and integrated rehabilitation services already exist in the health system. Furthermore, access to psychological support and community rehabilitation services were recommended areas for development in emergency response. The literature highlighted that underdeveloped rehabilitation services limit effective emergency response, and suggested that the existing unmet rehabilitation need, coupled with the new disaster need and subsequent media and political attention received, could provide a catalyst for the development of sustainable rehabilitation services, if advocacy efforts strong [6].

### Workforce

Developing the rehabilitation workforce is fundamental to any disaster response [55]. A significant barrier to providing rehabilitation services in disasters is the lack of rehabilitation professions [7, 56]. This review’s findings agree with the wider body of literature demonstrating poor awareness by professionals of the role they can play in disasters, and a lack of disaster specific training [54]. Rehabilitation 2030 [28] and the Sustainable development goal 3C [57] call for a substantial increase in health financing, recruitment, development, training and retention of the health workforce. More specifically, WHO calls for the expansion of rehabilitation workforce production and the strengthening of regulations and quality assurance mechanisms to upscale accessibility to quality rehabilitation services [58].

### Health information systems

The very limited evidence categorised under the health information systems building block is a symptom of the magnitude of challenges faced when attempting to collect quality data in disaster situations, when the focus is on saving lives in chaotic, austere situations [59]. Poor medical record keeping, the lack of assessment tools [54] and evidence on best practice in disaster contexts presents another barrier [60]. The challenge in conducting rigorous trials and collect data in complex and surprise disaster settings cannot be underestimated, and support and resources are needed to facilitate such projects. This may explain why there were no studies found on conflict, where the rehabilitation need is high and safety uncertain [61].

### Assistive technology

Our searches found no studies solely focused on assistive technology in disaster contexts, but 7 of the 27 included studies recommended the provision of assistive technology. The absence of focused literature is surprising given the high and growing levels of unmet need for assistive technology during crises [60]. This finding may be due to assistive technology sometimes being an afterthought of rehabilitation and lacking quality and appropriate prescription [62]. Provision of assistive technology in disaster contexts is limited by the lack of guidance on how to identify assistive technology needs in environments with little or no existing systems for provision [60]. However, it is encouraging that evidence of the need and provision of assistive technology in disaster contexts is emerging, and is supported by the United Nations Convention on the Rights of Persons with Disabilities formalizing the legal requirement to provide assistive technology to those who need it [63].

### Financing

It was unsurprising that this review found no studies that even mentioned the financing of rehabilitation or assistive technology in disaster contexts. Poor awareness of rehabilitation and assistive technology means that the development of services are rarely a government priority. Too often no coordination mechanisms or responsible officer exist, and consequently financing is limited or absent [64, 65].

### Leadership

Finally, the literature presents efforts in Iran, Nepal and the Philippines to create rehabilitation disaster response frameworks. However, the success, acceptability, and viability of progress with implementing these frameworks is yet to be evaluated in subsequent literature.

The secondary aim of the review was to summarize recommendations identified in the gathered literature. Early, multi-professional rehabilitation with assistive technology provision, that expanded into community services and included psychological support for patients were frequently recommended as priority areas for service delivery. Training for rehabilitation professionals to prepare them for disaster work was also highlighted often as a priority need. Strong advocacy efforts to create awareness of the need, the importance of rehabilitation in disasters, and the need of including rehabilitation in health strategy disaster preparedness and response, were consistently recommend across all studies.

In summary, the results from this scoping review suggest initiating actions to develop the following 4 priority areas; the provision of early and multi-professional rehabilitation, including assistive technology, psychological support, and integrated community services; disaster response specific training for rehabilitation professionals; advocacy efforts to create awareness of the importance of rehabilitation in disasters, and; the integration of rehabilitation into disaster preparedness and response. There are several resources [11–14, 28, 58, 66] and communities such as the WHO World Rehabilitation Alliance available to assist with implementing these recommendations.

### Limitations of this review

Relevant studies may have been missed from this review as only studies available in English could be considered, and not all reports are publicly available. In some instances, resources may not be available to publish details on activities. It would have been relevant to include country disaster management plans, but their identification would have been difficult as they would likely not be publicly available or, are published in languages other than English. Where rehabilitation services are yet to be developed within health system, rehabilitation is unlikely to feature in disaster management plans in any case. Another limitation is the exclusion of studies focused only on psychological rehabilitation, an important component of rehabilitation, as this is a highly specialized topic beyond our searches scope. The authors acknowledge that analysis according to categorization under one of the six WHO health system building blocks is imperfect as there will always been some overlap, such is the plexus of health system components. The methodology was chosen as a widely recognized and standardized method for health system analysis. Furthermore, categorizing the eligible studies and their recommendations together eases the readers understanding and aids clarity.

## Conclusion

The findings of this scoping review suggest that rehabilitation is poorly integrated into health systems disaster preparedness and response in LMICs, largely due to low awareness of rehabilitation, undeveloped existing rehabilitation health systems, as well as a lack of rehabilitation professionals, and disaster specific training for them. The lack of evidence demonstrating the rehabilitation and assistive technology need, and effective responses in disasters, is limiting advocacy efforts and the development of services in disasters. Reporting on the need and evaluation of responses to disasters from the field is essential for preparing feasible and meaningful preparedness plans.

Disasters can be an opportunity, even the catalyst, to develop rehabilitation within national health systems, and to integrate rehabilitation into disaster preparedness and emergency response plans. The main recommendations collected in this review suggest priority areas of actions to develop rehabilitation services in disasters.

### Supplementary Information

**Additional file 1. **Preferred Reporting Items for Systematic reviews and Meta-Analyses extension for Scoping Reviews (PRISMA-ScR) Checklist.

**Additional file 2: Appendix 2.** Bibliographic databases searches.

## Data Availability

The datasets used and/or analysed during the current study available in the appendix supplementary file.
