# Supplementary material for: Lessons learned from health system rehabilitation preparedness and response for disasters in LMICs: a scoping review
Source: BMC Public Health. 2024 Mar 14;24:806. doi: 10.1186/s12889-024-17992-2 (PMC10938837; doi:10.1186/s12889-024-17992-2)
Supplement: Supplementary file 2 — Additional file 2: Appendix 2. Bibliographic databases searches. [file 12889_2024_17992_MOESM2_ESM.docx]

**Appendix 2**

| **Bibliographic databases searches** |
| --- |
| PUBMED 10/05/22 = 2,268 results  (Rehabilitation [MeSH Terms] OR "assistive product*" OR "assistive technolog*" OR "assistive device*" OR (rehabilitation [MeSH Terms] AND burn) AND disaster[MeSH Terms] OR "humanitarian respons*" OR "humanitarian cris*" OR "humanitarian intervention*" OR "humanitarian action*" OR "armed conflict” Filters: from 2010 – 2022 |
| Scopus 10/05/22 = 2,041 results  TITLE-ABS-KEY ( humanitarian* OR "armed conflict" OR "natural disaster" OR disaster) AND (rehabilitation OR "wounds and injuries" OR "assistive technolog*") AND (( LIMIT-TO ( PUBYEAR , 2022 ) OR LIMIT-TO ( PUBYEAR , 2021 ) OR LIMIT-TO ( PUBYEAR , 2020 ) OR LIMIT-TO ( PUBYEAR , 2019 ) OR LIMIT-TO ( PUBYEAR , 2018 ) OR LIMIT-TO ( PUBYEAR , 2017 ) OR LIMIT-TO ( PUBYEAR , 2016 ) OR LIMIT-TO ( PUBYEAR , 2015 ) OR LIMIT-TO ( PUBYEAR , 2014 ) OR LIMIT-TO ( PUBYEAR , 2013 ) OR LIMIT-TO ( PUBYEAR , 2012 ) OR LIMIT-TO ( PUBYEAR , 2011 ) OR LIMIT-TO ( PUBYEAR , 2010 ) ) AND ( LIMIT-TO ( LANGUAGE , "English" )) |
| CINAHL*ALL CINHAL HEADINGS and exploded 10/05/22 = 2,015 results  (MH “Disaster+”) AND (MH “rehabilitation+” OR “MH “wounds and injuries+” OR MH “assistive technology devices+”)  - from 2010, English only. |
| Cochrane *ALL MESH TERMS 10/05/22 = 3 results  Title/abstract/keyword, explode all trees  Reviews only published between January 2010 - May 2022  (Rehabilitation OR “self help devices” OR “wounds and injuries”) AND (disasters OR natural disasters OR armed conflicts OR relief work) |
